# Multiscale Modeling and Characterization of Graphene Epoxy Nanocomposite

**DOI:** 10.3390/polym16091209

**Published:** 2024-04-26

**Authors:** Collins Ekeowa, SD Jacob Muthu

**Affiliations:** Faculty of Engineering and Applied Science, University of Regina, Regina, SK S4S 0A2, Canada; collins.ekeowa@gmail.com

**Keywords:** polymer nanocomposites, interphase properties, multiscale modeling, molecular dynamics

## Abstract

This study aims to characterize graphene epoxy nanocomposite properties using multiscale modeling. Molecular dynamics was used to study the nanocomposite at the nanoscale and finite element analysis at the macroscale to complete the multiscale modeling. The coupling of these two scales was carried out using the Irving–Kirkwood averaging method. First, the functionalization of graphene was carried and 6% grafted graphene was selected based on Young’s modulus and the tensile strength of the grafted graphene sheet. Functionalized graphene with weight fractions of 1.8, 3.7, and 5.6 wt.% were reinforced with epoxy polymer to form a graphene epoxy nanocomposite. The results showed that the graphene with 3.7 wt.% achieved the highest modulus. Subsequently, a functionalized graphene sheet with an epoxy matrix was developed to obtain the interphase properties using the MD modeling technique. The normal and shear forces at the interphase region of the graphene epoxy nanocomposite were investigated using a traction-separation test to analyze the mechanical properties including Young’s modulus and traction forces. The mean stiffness of numerically tested samples with 1.8, 3.7, and 5.6 wt.% graphene and the stiffness obtained from experimental results from the literature were compared. The experimental results are lower than the multiscale model results because the experiments cannot replicate the molecular-scale behavior. However, a similar trend could be observed for the addition of up to 3.7 wt.% graphene. This demonstrated that the graphene with 3.7 wt.% shows improved interphase properties. The macroscale properties of the graphene epoxy nanocomposite models with 1.8 and 3.7 wt.% were comparatively higher.

## 1. Introduction

Polymers are extending the horizons of designers in all branches of engineering owing to several advantages including a high strength-to-weight ratio, ease of manufacturing with improved corrosion control, etc. [1]. Therefore, polymers have been used in several engineering applications including automobiles, aircraft components, construction, etc. [2]. However, with inferior mechanical properties compared with metals and alloys [3], polymers require strengthening mechanisms using nanofillers to improve their properties, thus creating polymer nanocomposites. Different combinations of nanofillers (graphene, carbon nanotubes, cellulose, silica, etc.) have been used to produce nanocomposites, and graphene–polymer nanocomposites have ignited great interest owing to graphene’s remarkable properties [4].

Graphene is a monolayer structure of carbon atoms covalently bonded to form one of the strongest bonds, which has resulted in excellent mechanical and physical properties [5] and has made graphene a favorable reinforcement for improving polymer properties. For instance, epoxy reinforced with graphene resulted in improved properties of graphene epoxy nanocomposites [6,7], and the characterization has shown that the strength of the interphase region between a graphene sheet and the epoxy matrix dictates the macro properties of the nanocomposite material. Further, reviews of graphene-reinforced composites have shown that the addition of graphene and its derivatives greatly improves the mechanical and structural properties of the polymer materials [8,9]. As the reinforcements reach the nanometer level, the interactions of nanofillers with the matrix at the interphase region largely improve and thus enhance the nanocomposite properties [10,11]. In this context, the surface area/volume ratio of nanofillers is crucial to the understanding of their structure–property relationships. Further, the dispersion of the nanofillers promotes the interfacial contact of the nanoparticles with the polymer matrices and subsequently improves the interphase properties [12]. Hence, understanding the structure–property relationships and the influence of the interphase region on the macroscopic properties is necessary for tailoring polymer nanocomposites to particular applications [13,14]. In addition, the functionalization of nanoparticles with precise chemical structure is expected to improve the compatibility of nanoparticles for different types of polymer matrices, which could further improve the interphase region and the overall properties of the nanocomposites [15,16,17].

Based on the above review, it can be concluded that nanocomposite properties are influenced by the properties of the interphase region, which further depend on the interfacial adhesion between the matrix and nanofillers and also the types of functionalization. The interphase region properties are unique and differ from the matrix and reinforcement. In addition, the interfacial adhesion can also be affected by the dispersion of the nanofillers within the matrix. If the nano-reinforcements are poorly dispersed, they can lead to agglomeration and further reduce the overall properties of the nanocomposites [18]. Furthermore, the weight fraction and alignment of nanofillers can also affect the properties of nanocomposites [19]. Hence, a clear understanding of the interphase region and the factors affecting interphase properties would help in improving and tailoring nanocomposite properties for their required applications.

Owing to the complexity of the interphase region, a combination of both macro-and nanoscale analyses is required for better characterization [20]. However, experimental analysis generally focuses on macro-scale properties and is less efficient in providing the intricacy of the nanoscale properties within the interphase region [21]. The limitations in length and time scales in experimental analyses have led to numerical modeling such as finite element analysis (FEA) at the macroscale and molecular dynamics (MD) at the atomic scale being utilized [22]. These numerical methods can model nanocomposites and their interphase region properties at different lengths and time scales, and the coupling of different lengths and time scales is generally known as multiscale modeling (MSM).

Over the past decade, various multiscale modeling methods such as adaptive, heterogeneous, and quantum mechanics coupled with molecular mechanics (QM–MM) have been developed to address problems involving different lengths and time scales [23]. The adaptive mesh refinement method was used to analyze crack propagation at different lengths and time scales [24] and also to model dynamic and turbulent regions without affecting the precision of the solution, but it is limited to pre-determined measured computational grids. QM–MM combines the strengths of the QM (accuracy) and MM (speed) approaches [25]. However, the limitation in focusing on smaller simulation regions constrains the application of the QM–MM method to broader applications. Combining MD simulations (nanoscale) with finite element analysis (FEA) at the macroscale was also considered within the multiscale domain. However, passing the MD boundary conditions to macroscale analysis results in inaccuracies owing to changes in the computational domain size [26]. To circumvent the limitations mentioned above in computational domain size and the transfer of boundary conditions from macro to nanoscale models, heterogeneous multiscale methods (HMM) became popular. HMM follows a top-down strategy using the Irving–Kirkwood averaging method. The basic starting point of HMM is an incomplete macroscale model, and the nanoscale model is used as a supplement to supply missing data, including boundary conditions from interphase region analysis [27].

Hence, the objectives of this research are to characterize the interphase region properties of graphene epoxy nanocomposite using molecular dynamics based on weight fractions, functionalization, and the interfacial adhesion (cross-linking) between the epoxy and graphene reinforcement. Further, the MD-coupled FEA graphene epoxy nanocomposites reference volume element (RVE) is characterized to obtain the elastic properties of the nanocomposites. Finally, the results are validated using the available experimental results. 

## 2. Materials and Methods

**Figure 1:**
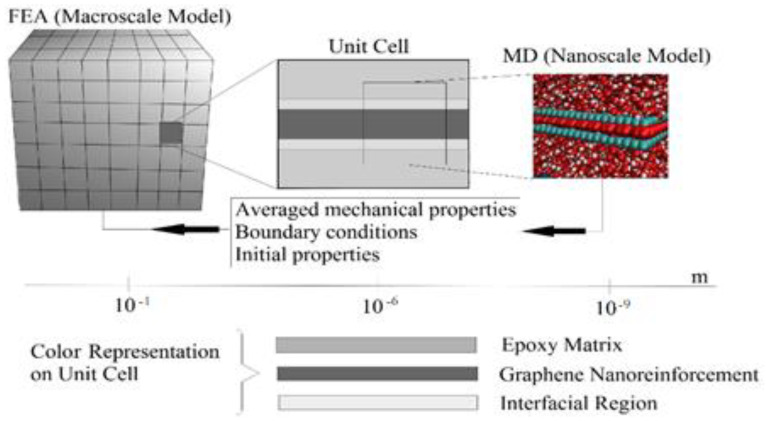
Multiscale modeling computational procedure.

Figure 1 summarizes the computational sequence of the multiscale analysis. A unit cell was extracted from the FEA macroscale model, which consists of an epoxy matrix, a graphene nano-reinforcement, and the interfacial region. These individual constituents were modeled and analyzed using molecular dynamics (MD) simulation to obtain the interfacial region properties, which were then transferred to the FEA macro model to obtain the nanocomposite macro properties. 

First, MD modeling of graphene functionalized with 3%, 6%, and 9.8% OH and COOH functional groups was completed. Then, functionalized graphene with improved bonding properties was acquired. The better bonding properties were determined by the valence of the oxygen atoms attached to the functionalized graphene. Subsequently, MD modeling of the functionalized graphene with epoxy matrix was carried out to obtain the interphase properties. The different weight fractions of graphene were attached to the epoxy matrix, and the best weight fraction was obtained by checking the cross-linking between the graphene and the epoxy matrix. The MD model of the graphene epoxy nanocomposites was then tested to obtain the interphase properties by applying a displacement (normal and shear direction) to the graphene sheet. As the displacement was applied, the traction forces were obtained to characterize the interfacial properties of the nanocomposites. The MD-modeled nanocomposite properties were then transferred to the macroscale model by coupling the two scales. The coupled models were then analyzed to obtain the elastic properties of the graphene epoxy nanocomposite. The results were then validated using the available literature. 

### 2.1. Multiscale Modeling Theory

#### 2.1.1. MD Modeling

The MD modeling was carried out using the open-source LAMMPS software package [28]. A unit cell was defined to describe the molecular interactions of atoms. The atoms within the unit cell have both bonded and non-bonded interactions, hence the total potential energy V(r) is represented by

(1) $$V\left(r\right)=V_{bonded}+V_{non-bonded}$$

The bonded interactions occur owing to the covalent bonds between nano-reinforcement and matrix atoms within a unit cell. The bonded interactions are calculated using the Optimized Potentials for Liquid Simulation (OPLS), which is the sum of all the bonded interaction energies (*V_bonded_*), as defined below;

(2) $$\begin{array}{c} V_{bonded}=\sum [K_{r}{\left(r-r_{0}\right)}^{2}+K_{\theta }{\left(\theta -\theta_{0}\right)}^{2}+\frac{W_{1}}{2}\left[1+\cos(\phi -\phi_{1}\right]+\frac{W_{2}}{2}\left[1+\cos(2\phi -\phi_{2}\right]\\ +\frac{W_{3}}{2}\left[1+\cos(3\phi -\phi_{3}\right]+\frac{W_{4}}{2}\left[1+\cos(4\phi -\phi_{4}\right]] \end{array}$$

where

- *K_r_* is the bond constant,
- *r* is the bond length,
- *K_θ_* is the angle energy constant,
- *θ* is the actual bond angle,
- *θ*_0_ the reference bond angle,
- *W* is OPLS dihedral constants,
- *Φ* is an equilibrium angle.

The non-bonded interactions consist of two potential functions, the van der Waals potential for short interactions and electrostatic potential for the atoms within one electric field (long-range interactions), and are defined by

(3) $$V_{non-bonded}=V_{\mathrm{van}\mathrm{der}\mathrm{Waals}}+V_{electrostatic}$$

The electrostatic interactions go beyond the interaction range, hence only van der Waals interactions are considered for MD simulations. The van der Waals interaction is given as the Lennard–Jones (LJ) potential and obtained as follows:

(4) $$V\left(r_{ij}\right)=4\xi \left[{\left(\frac{\sigma_{ij}}{r_{ij}}\right)}^{12}-{\left(\frac{\sigma_{ij}}{r_{ij}}\right)}^{6}\right]$$

where

- ξ is the measurement of the Van der Waals attraction between the united atoms,
- $\sigma_{ij}$ is the measurement of the distance between two non-bonded united atoms,
- *r_ij_* is the distance of separation between united atoms (${r_{ij}}^{12}$ is the short-range attraction and ${r_{ij}}^{6}$ is the long-range attraction),
- $\frac{\sigma_{ij}}{r_{ij}}$ represents the repulsive properties of the united atoms (steep side of the curve),
$-\frac{\sigma_{ij}}{r_{ij}}$ represents the attraction properties of the united atoms (smooth side of the curve).

The LJ potential is used to define the motion and interaction of atoms for both bonded and non-bonded interactions within a composite unit cell.

#### 2.1.2. Boundary Conditions and Force Calculations

**Figure 2:**
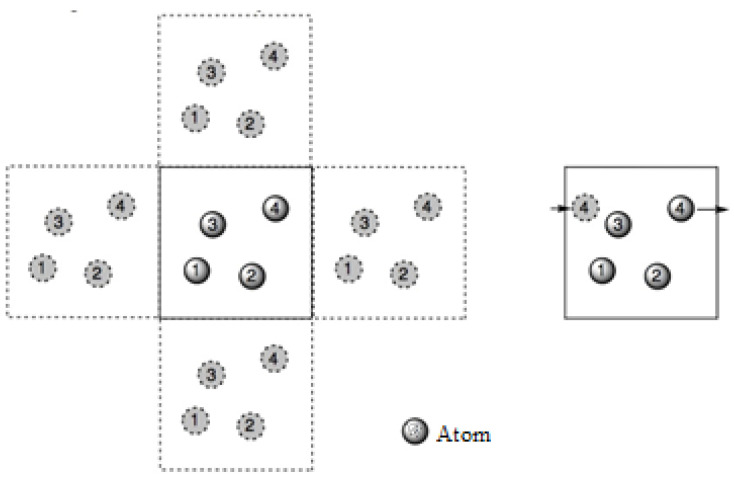
Periodic boundary conditions and the particle trajectories in the central simulation box.

Since the motions and interactions of atoms can alter the size and the force calculations within a unit cell, boundary constraints are essential for maintaining the size of the unit cell and also for controlling the atomic interactions. Therefore, periodic boundary conditions are set within the unit cell, which states that an atom moves out of a unit cell and will appear on the adjacent unit cell, as shown in Figure 2.

Ideally, every atom interacts with every other neighbouring atom within a cut-off distance. To define the cut-off distance, the coordinates of the individual atoms and their movement path (trajectories) within a unit cell are required. Since the interatomic potential is used to define the force acting on atoms, the interatomic potential is integrated using Verlet integration to obtain the required coordinates and trajectories [29]. Using the ensemble of the atoms’ position vectors $r_{i}\left(t\right)=r_{1}\left(t\right)\ldots r_{N}(t)$, the interatomic potential can be used to define the force as follows:

(5) $$f_{i}=-\nabla V=m_{i}\frac{d^{2}r_{i}}{dt^{2}}$$

where

- *t* is the time,
- $f_{i}$ are the forces acting on ith atom,
- *m_i_* is the mass of ith atom,
- *r_i_* is the position vector,
- $a_{i}=\frac{d^{2}r_{i}}{dt^{2}}$.

Utilizing the Taylor series, the Verlet algorithm uses a position vector $r_{i}(t)$, $r_{i}(t-∆t)$, and acceleration (*a*_i_) at time t to calculate the new position vector $r_{i}(t+∆t)$ and velocities $v_{i}(t)$ and $v_{i}(t+\frac{1}{2}∆t)$ at time t and $\left(t+\frac{1}{2}∆t\right)$, respectively, and the corresponding equations are given below.

(6) $$r_{i}\left(t+∆t\right)\approx 2r_{i}\left(t\right)-r_{i}\left(t-∆t\right)+a_{i}(t){\left(∆t\right)}^{2}\;v_{i}\left(t\right)\approx \frac{r_{i}\left(t+∆t\right)+r_{i}\left(t-∆t\right)}{2∆t}\;v_{i}\left(t+\frac{1}{2}∆t\right)\approx \frac{r_{i}\left(t+∆t\right)+r_{i}\left(t\right)}{∆t}$$

Since the change in velocity depends on the change in temperature of the system, the Verlet integration is performed within the constant number of atoms (*N*), volume (*V*), and temperature (*T*) ensemble. However, for the stabilization of velocity or acceleration of atoms, the maximum temperature should reach a value of approximately 300 or 400 *K*. To achieve the required temperature, a Nose–Hoover thermostat (NHT) algorithm is introduced. Within this algorithm, a friction dynamic variable ξ is added, which slows down or accelerates atoms until the maximum temperature is reached, as given in the equation below:

(7) $$\xi \left(t+\delta t\right)=\xi \left(t+\frac{dr(t)}{t}\right)+\frac{dr(t)}{Q}\left[\sum_{i=1}^{N}\frac{v_{i}{\left(t+\frac{dr(t)}{2}\right)}^{2}}{2}-\frac{3N+1}{2}k_{B}T\right]$$

where

- *Q* is the effective mass of the system associated with ξ,
- *T* denotes the target temperature,
- $\frac{3N+1}{2}k_{B}$ represents the kinetic energy of the united atoms within the unit cell.

The Verlet algorithm gives the trajectories and coordinates of the atoms within the MD simulation, while the NHT algorithm maintains the accuracy of the simulation results.

### 2.2. Macroscale Modeling

The macroscale model was defined using FEA, and the displacements u, v, and w of an isotropic material are used in terms of Cartesian x, y, and z coordinates. From Hook’s law, the overall response of the macroscale model can be defined as

(8) $$\sigma_{ij}=C_{ijkl}\varepsilon_{kl}\;[7]$$

where i, j, k, l = x, y, z, and the three-dimensional stress (σ)/strain (ε) law for the macroscale model are given by

(9) $$\sigma_{xx}=\frac{E}{\left(1+v)(1-2v\right)}\left[\left(1-v\right)\varepsilon_{xx}+v(\varepsilon_{yy}+\varepsilon_{zz})\right]\;\sigma_{yy}=\frac{E}{\left(1+v)(1-2v\right)}\left[\left(1-v\right)\varepsilon_{yy}+v(\varepsilon_{yy}+\varepsilon_{zz})\right]\;\sigma_{zz}=\frac{E}{\left(1+v)(1-2v\right)}\left[\left(1-v\right)\varepsilon_{zz}+v(\varepsilon_{yy}+\varepsilon_{yy})\right]$$

where

- $C_{ijkl}$ is the compliance matrix,
- *E* is the elastic modulus,
- *v* is the Poisson’s ratio.

The displacements of the nanocomposite macroscale model can be obtained from the above equations.

### 2.3. Coupling of MD with FEA

**Figure 3:**
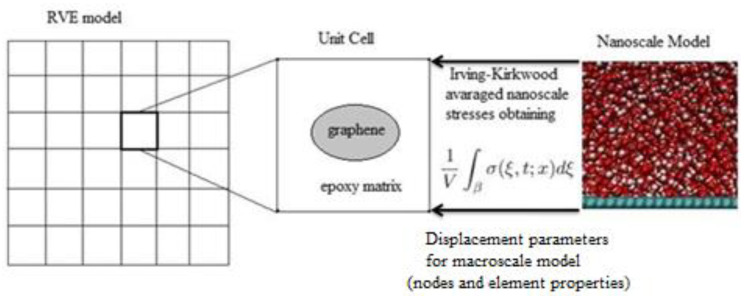
Coupling of molecular dynamics with finite element analysis.

A schematic of the coupling process is shown in Figure 3. To achieve this, the atom positions and trajectories from the nanocomposite model were averaged using the Irving–Kirkwood method and were used to calculate the displacement and momentum using the Verlet integration formulating the macroscale unit cell. 

The next step was to assign boundary conditions, which were used to define the degrees of freedom of the macroscale unit cell nodes and elements based on the nanoscale model properties. From the nanoscale model, the mass and velocity in addition to the position vectors were extracted from the Verlet integration to calculate momentum, as per the equations given below:

(10) $$\frac{d}{dt}\sum_{i=1}^{N}q_{i}(r_{i},t)=\sum_{i=1}^{N}m_{i}\frac{dv}{dt}$$

where

- *r_i_* represents a set of molecules defined in terms of their time-dependent coordinates,
- *q_i_* is the momentum.
- Using $q_{i}=m_{i}\frac{dr_{i}}{dt}$, the time evolution of momentum from the nanoscale to the macroscale was obtained using the equation given below:
  (11) $$\frac{d}{dt}\sum_{i=1}^{N}q_{i}\left(t\right)v(r_{i}\left(t\right),r)=\sum_{i=1}^{N}\left[q_{i}\frac{dr_{i}}{dt_{i}}\frac{dv_{i}}{dr_{i}}+\frac{dq_{i}}{dt}v_{i}\right]$$

This resulted in the attainment of the momentum equation assigned to the nodes in the macroscale model, thereby giving a rotational degree of freedom to the nodes and elements represented by

(12) $$q_{i}\left(r_{i,}t\right)=\sum_{i}m_{i}v_{i}(r-r_{i}\left(t\right))$$

where

- *(r−r_i_(t))* represents the displacement of the united atoms with respect to time,
- *m_i_* is the mass of the atom I,
- *v_i_* is the velocity.

In addition, the boundary conditions of deflection and stresses (defined by force) to the macroscale nodes and elements were extracted from the nanoscale model. These deflections and stresses were defined within the displacement matrix, where the force (normal or shear) was equivalent to a translation degree of freedom in the macroscale unit cell. The properties of the nodal displacements of matrix and element nodal forces were derived from the Irving–Kirkwood stress formula, as given below:

(13) $$\sigma \left(\xi ,\;t;r\right)=-\sum_{i}^{N}\left(m_{i}v_{i}⊗v_{i}\right)\delta \left(r_{i}-\xi \right)-\frac{1}{2}\sum_{j\neq i}\left(r_{i}-r_{j}\right)⊗f_{ij}\int_{0}^{N}\delta \left(\lambda r_{i}+\left(1-\lambda \right)r_{j}-\xi \right)d\lambda$$

where

- *f_ij_* represents the force acting on the i_th_ atom by the j_th_ atom, defining the translation DOF in the macroscale unit cell,
- *δ* is the Dirac delta function
- *σ* represents the stress ($\sigma_{ij}=c_{ijkl}\varepsilon_{kl}$).

This aligned the properties assigned to the elements and nodes in the macroscale model with the nanoscale model properties, thereby completing the coupling of the molecular dynamics model with the macroscale model for the nanocomposite.

## 3. Modeling Procedure

The MD analysis procedure explained in the previous paragraphs was used to model the constituents of the graphene epoxy nanocomposites. First, the matrix was modeled, followed by the nano-reinforcement, and finally the interfacial region to study the interphase region’s mechanical properties.

### 3.1. Matrix Modeling

**Figure 4:**
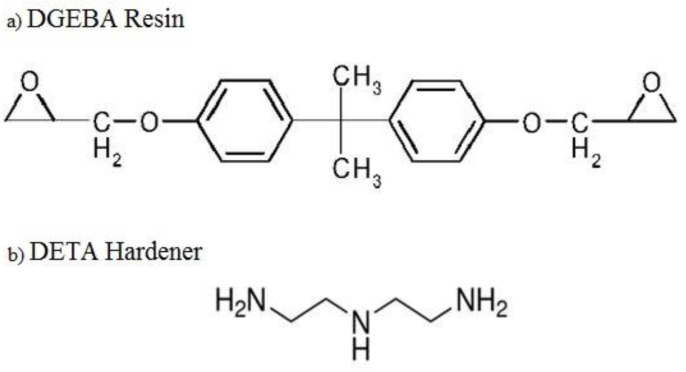
(**a**) Molecular structure of the DGEBA resin; (**b**) molecular structure of the DETA hardener.

For the current study, the epoxy EPON 862: Diglycidyl Ether Bisphenol A (DGEBA) was selected as the matrix and diethylenetriamine (DETA) as a hardener. The basic atomic structures of the resin and the hardener are shown in Figure 4. The epoxy and hardener chemical reaction was modeled as a non-bonded interaction. Hence, the Lennard–Jones (LJ) potential was used to define the non-bonded interactions. The resin and hardener atoms were grouped as one united atom and were modeled using the optimized potential for liquid simulations (OPLS) force field. The LJ potential within the non-bonded energy introduces the van der Waal interactions between the individual atoms (CH_3_, CH_2_, CH, NH_2_, NH, and alkyl groups) of the resin and hardener. This combination was modeled as one united atom corresponding to the masses of the individual constituents. This simplification reduces the computation time of the LAMMPs code.

First, an epoxy unit cell with a stoichiometric ratio of two molecules of resin and one molecule of hardener (2:1) was developed. This unit cell contained a total of 117 atoms. Their initial coordinates and the details for bonds, angles, and dihedrals were written in LAMMPS, and periodic boundary conditions were applied in all directions. The use of the united atoms concept reduced the total atoms of a unit cell from 117 individual atoms to 83 united atoms. Furthermore, owing to the difference in mass of each atom within the resin and hardener system, the 83 united atoms were reduced to 52 united atoms. This reduced the resin and hardener epoxy system to approximately 31 resin and 21 hardener united atoms. Therefore, the epoxy unit cell contained a total of 52 united atoms. The modeling is summarized in Table 1.

**Figure 5:**
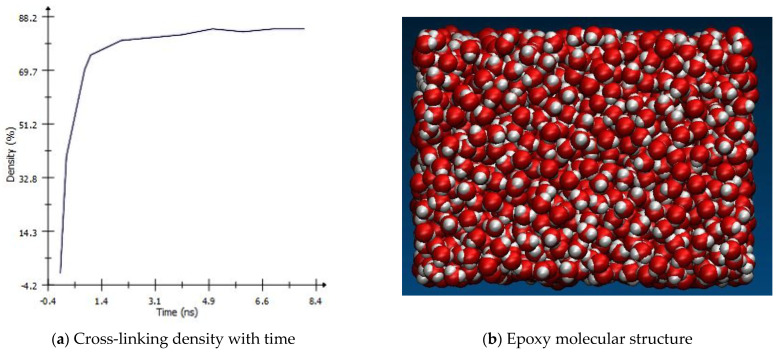
Cross-linking of DGEBA epoxy with DETA hardener.

Within one united atom, the cross-linking of resin and harder occurs at a controlled constant temperature, which was equilibrated using the Nose Hoover thermostat (NVT) method. This equilibration was run (NVT) at 1000 K and a low density of 0.6 g/cm^3^. However, to maintain the required weight fraction, the simulation box will be reduced using the NVT method for 100 femtoseconds (fs) at 450 K with a constant density of 0.9 g/cm^3^. In addition, the maximum atom movements were limited to 0.2 Å to smooth the energy changes during the simulation. The cross-linking reaction of hardener and resin density was recorded with time and is shown in Figure 5a. The figure shows that the cross-linking quickly increases in the early stages of the simulation and then slows down while the network grows continuously. This shows that the perfect cross-linking of resin to hardener was achieved. The resultant MD model of the epoxy matrix is shown in Figure 5b. The cross-linked network had a density of 1.12 g/cm^3^ at low pressure of around 1 atm, which is in close agreement with the result shown by Li et al. [30]. This epoxy model was then reinforced using functionalized graphene nano-reinforcement.

### 3.2. Modeling of Nano-Reinforcement

The nano-reinforcement of graphene was modeled using LAMMPS code. The bonded interactions between carbon–carbon atoms were modeled using Tersoff and optimized Tersoff potentials. The non-bonded van der Waals interactions between individual carbon atoms at different atomic planes were modeled using Lennard–Jones (LJ) potentials. To satisfy the bulk density of graphene (2.2 g/cm^3^), the equilibrium distance between each carbon atom was set to 3.4 Å as part of the initial conditions of MD simulation, and the initial setup is summarised in Table 2.

**Figure 6:**
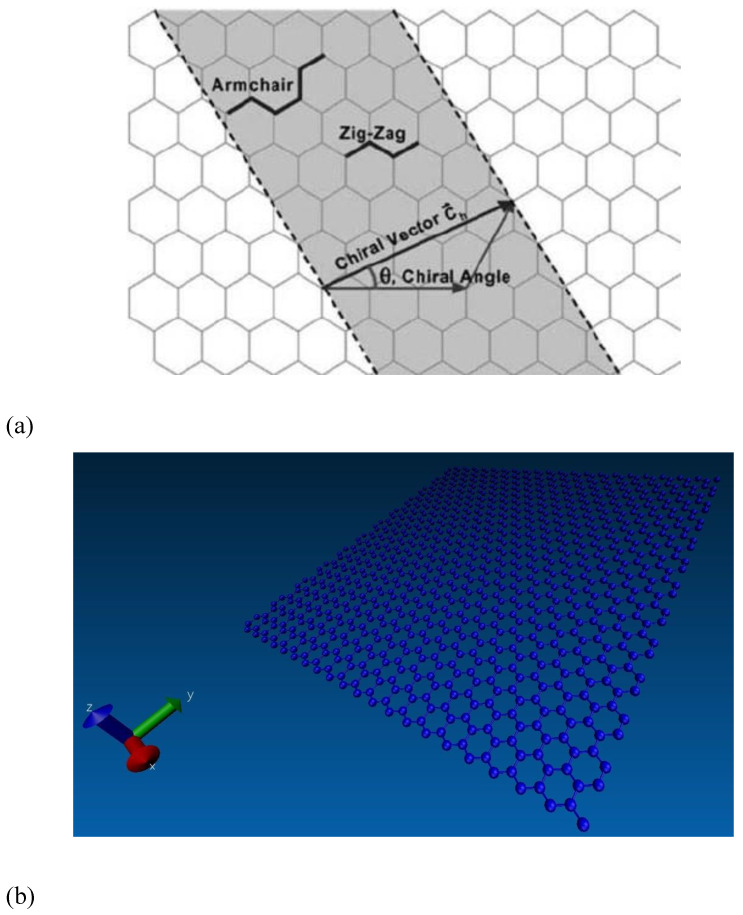

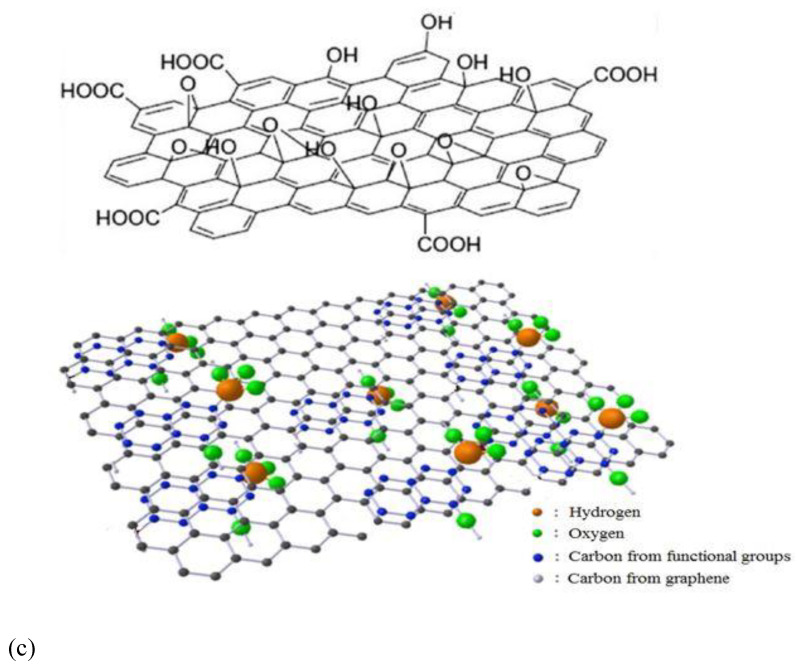
Graphene modeling and functionalization. (**a**) Schematic diagram of a hexagonal graphene sheet [31]. (**b**) Graphene LAMMPS Model. (**c**) Functionalized graphene sheet.

The graphene sheet size was set at 5 nm along the x-direction and 10 nm along the y-direction for matching with a 2:1 stoichiometric ratio after blending with the epoxy matrix. The chirality angles of graphene were equal to 0° and 30° for armchair and zigzag configurations, respectively, as shown in Figure 6a. Here, the periodic boundary conditions (PBC) were applied along the x direction of the graphene edge to remove the finite length effect. The free boundary conditions were applied along the y direction. The applied boundary conditions for preparing the graphene sheet for proper cross-linking with the epoxy matrix and the LAMMPS model are shown in Figure 6b.

### 3.3. Interphase Region Modeling

To produce a good interphase region and interfacial adhesion, the graphene sheet needs to be functionalized. The functional groups of OH and COOH were added onto the edges and to the surface of the graphene sheet in the LAMMPS model. The graphene sheet has two types of edges, such as zigzag and armchair, and the functional groups pair with them differently. Each carbon atom on the zigzag edge has an unpaired electron, making it easy to bond with the COOH and OH functional groups. However, at the armchair edge side, the carbon atoms are more stable due to the presence of triple covalent bonds, hence the functional groups leave a valence of oxygen atoms at the edges during functionalization.

These functional groups were randomly grafted onto the 32 carbon atoms from the graphene layer using the LAMMPS code. The influence of grafting density on the mechanical properties of graphene was studied by grafting 3.0%, 6.0%, and 9.8% of functional groups to the graphene sheet. The approximate number of atoms added to the graphene sheet by the functional groups is given in Table 3. Based on the interfacial region properties, the effect of the distribution of the COOH and OH functional groups on the graphene sheet showed optimal functionalization at 6.0% of functional groups. A further increase in functionalization to 9.8% distribution showed saturation and no improvement of properties. Thus, a functionalized graphene sheet for reinforcement of the epoxy matrix will have 46 atoms (6% functional groups). Further, these 46 atoms within the graphene system have similar interactions and properties (covalent bonds of COOH, OH, and one valency oxygen atom), hence they are defined as one united atom. The functionalized graphene model is shown in Figure 6c.

The functionalized graphene sheets were then used to develop the graphene epoxy nanocomposite models with one, two, and three graphene sheets which represented 1.8, 3.7, and 5.6 wt.% respectively. The weight fraction of the graphene was calculated using the total number of united atoms modeled in the LAMMPS code. For example, a functionalized graphene sheet consists of one united atom, whereas the epoxy resin consists of 52 united atoms. Thus adding one graphene sheet (1 united atom) with 52 united atoms of epoxy gives a weight fraction of 1.8 wt%, as given below.

(14) $$\frac{\mathrm{Total}\mathrm{number}\mathrm{of}\mathrm{united}\mathrm{atoms}\mathrm{in}a\mathrm{Functionalized}\mathrm{graphene}\mathrm{sheet}}{\mathrm{Total}\mathrm{number}\mathrm{of}\mathrm{united}\mathrm{atoms}\mathrm{in}\mathrm{Epoxy}-\mathrm{graphene}\mathrm{composite}}\times 100=\mathrm{Weight}\mathrm{fraction}\;\frac{1}{52+1}\times 100=1.8\;wt\%$$

**Figure 7:**
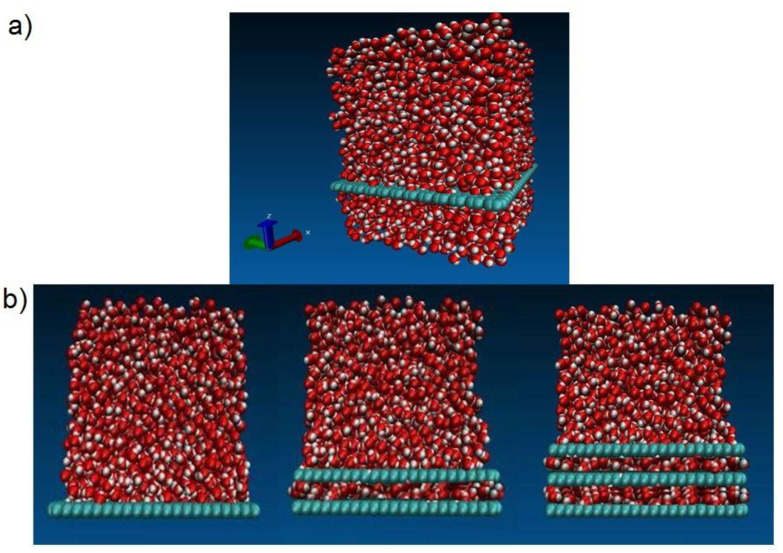
(**a**) 3D model for the graphene epoxy nanocomposite; (**b**) configurations of 1, 2, and 3 graphene sheets within the graphene epoxy nanocomposite.

Based on the above calculation, the nanocomposites with three weight fractions (1.8, 3.7, and 5.6 wt.%) of graphene were modeled (Table 4). The graphene sheets were stacked normally to the loading axis (x-axis) and parallel to each other during the modeling process. The free oxygen atoms within the functionalized graphene sheet formed covalent bonds with carbon atoms of the epoxy while developing the nanocomposite model. This mimics a strong interphase region bond between the graphene and epoxy matrix. The average spacing (cutoff distance) between the two individual constituents of united atoms was set to approximately 2 Å. If the cutoff distance is not within the limit, the covalent bonds between the united atoms will be broken and thus reduce the bonding strength. Therefore for better interfacial bonds within the nanocomposites unit cell, the interatomic forces between the constituent’s united atoms would be minimized to maintain the cutoff distance. This was achieved by pulling the united atoms together using the conjugate gradient method and thereby improving the interfacial bond. The size of the unit cell for the different weight fractions of graphene reinforcement configurations is listed in Table 4, where the unit cell dimensions are represented by length (a), breath (b), and width (c). Figure 7a shows a 3D model of a graphene epoxy nanocomposite structure and Figure 7b shows the 1, 2, and 3 graphene sheet configurations within the graphene epoxy nanocomposite.

The next step was to define the interactions (bonding characteristics) between the united atoms of the functionalized graphene and epoxy within the nanocomposite unit cell. This modeling process was carried out using an NVT-based molecular dynamic ensemble with 5000 time steps at a temperature of 300 K. The time step size was maintained at 0.8–1.0 femtosecond (fs), and the cut-off distance was 2 Å. The positions and velocities of the united atoms were updated using a Verlet integration scheme [32] for every time step. As the modeling analysis progressed, the united atoms’ positions changed owing to the temperature change; therefore, the Verlet integration update is essential for positioning the plane of the graphene sheets perpendicular to the loading axis. To control the graphene weight fraction in the system, the graphene sheets were added one by one. After the addition of each sheet, the Verlet integration scheme was used to update the positions and the velocities of the united atoms to determine the interaction and bond strength.

### 3.4. Interphase Region Properties

To obtain the interphase region properties, both normal and shear traction separation numerical experiments were conducted by slowly displacing the graphene sheet under displacement control within the unit cell in the normal and shear directions. As specified before, the cut-off distance between the epoxy and graphene sheet united atoms was maintained at 2 Å, and the debonding cutoff distance was kept at 20 Å. Beyond this value, the graphene sheet starts debonding from the epoxy matrix. The boundary conditions for both normal and shear traction numerical experiments were non-periodic along the z-axis and non-periodic along the xy plane.

**Figure 8:**
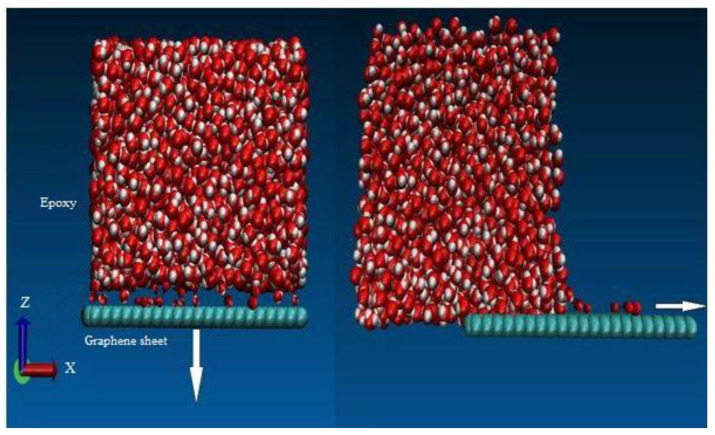
Normal and shear displacement of the graphene sheet in graphene epoxy nanocomposite.

The rate of loading for the normal traction separation (pullout) experiment was 0.01 Å/fs for a period of 3 fs, and for the shear traction separation (cohesive) experiment it was 0.001–0.0001 Å/fs for a period of 1.5 fs. The positions and velocities of the united atoms for each time step were updated using the Verlet time integration scheme. The tested model after final failure (beyond 20 Å) is shown in Figure 8.

As the graphene sheet was pulled, the reaction force (f_ij_) was developed at the interface between united atoms owing to the nonbonded interactions (Lennard–Jones (LJ) potentials) by which the force versus displacement plots were obtained. This induced reaction force causes the change in bond stretching, bond angle, and dihedral within the optimized potentials for liquid simulations (OPLS), which were defined by the total potential energy, V(r) (refer to Equation (1)), of the nanocomposite system. Then, the elastic constants (C_ij_) for the MD model were obtained using the second derivative of the total potential energy (OPLS) with respect to the lateral strain components, as given in the equation below:

(15) $$C_{ij}=\frac{1}{V}\left(\frac{\partial^{2}V(r)}{\partial \varepsilon_{i}\partial \varepsilon_{j}}\right)\;\sigma_{ij}=\sum_{i=0}^{6}C_{ij}\varepsilon_{ij}$$

where

- $\varepsilon_{i}$ and $\varepsilon_{j}$ are strain components,
- V(r) is potential energy,
- V is simulation cell volume,
- $\varepsilon_{ij}$ represents strain.

The elastic constants (C_ij_) for the united atoms i and j can be written as an elastic matrix. This is a 6 × 6 matrix, which describes the stress–strain behaviour of the nanocomposite and the elastic constant coefficients within it. For the graphene epoxy nanocomposite system, the elastic moduli were calculated using the following equations:

(16) $$\text{Young’s}\mathrm{modulus}\left(E\right)=\mu \left(\frac{3\lambda +2\mu }{\lambda +\mu }\right)\;\mathrm{Bulk}\mathrm{modulus}\left(K\right)=\lambda +\frac{2}{3}\mu \;\mathrm{Shear}\mathrm{modulus}\left(G\right)=\mu \;\text{Poisson’s}\mathrm{ratio}\left(v\right)=\frac{\lambda }{2(\lambda +\mu )}$$

where λ is the Lamé coefficient.

All the properties of the interfacial region are calculated within the LAMMPS code and then tabulated to obtain the overall mechanical properties of the graphene epoxy nanocomposite materials. Finally, when the bond stretching energy of the united atoms reached a point closer to the failure (beyond 20 Å), the united atoms were debonded and the associated angles and dihedral were deleted, resulting in separation along both the normal and shear directions. These displacement and stress properties were then used in the coupling of molecular dynamics modeling with finite element analysis.

## 4. Macroscale Model Analysis Procedure

**Figure 9:**
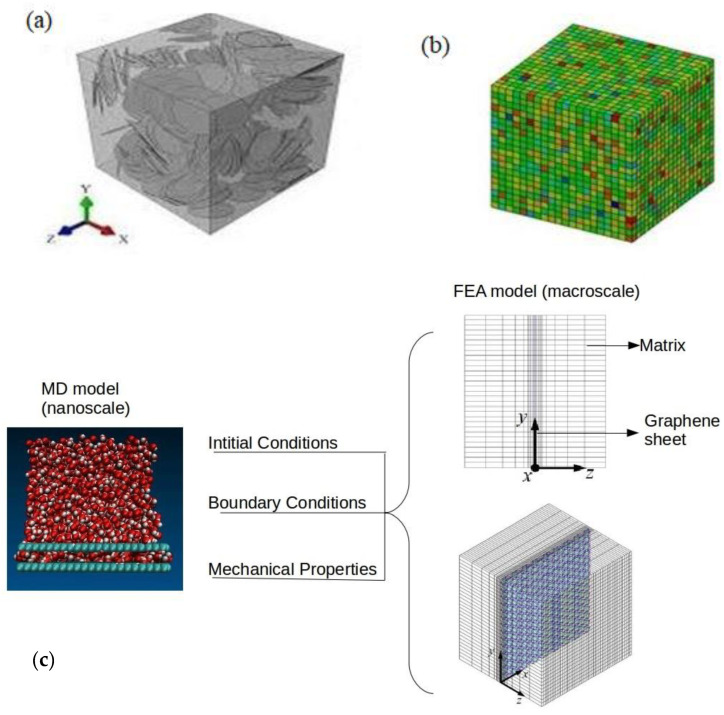
(**a**) Three-dimensional cubic RVE filled with graphene nano-reinforcement with an aspect ratio of 100. (**b**) Flexible meshing of RVEs; each disc was partitioned into four symmetric parts. (**c**) Initial and boundary conditions from the MD scale to the macroscale (FEA).

The FEA simulations were performed to calculate the nanocomposite mechanical properties using representative volume elements (RVEs) of the structure. As explained before, the FEA macroscale model was obtained using parameters acquired from the nanoscale model, such as the initial and boundary conditions, which were used to formulate the coordinate systems for the macroscale model nodes and elements, and the displacements from the nanoscale model were used to conduct the static FEA experiment (Figure 9c). The number of elements within the macroscale RVEs was set to 100, and the sizes of the RVEs were adjusted based on the averaged weight fraction of the nano-reinforcement from MD analysis. To accurately model the desired weight concentration of graphene to epoxy matrix inside the RVEs, the models were constructed to satisfy the periodicity criterion (similar to periodic boundary conditions). This means that if a filling particle passes one boundary side of the RVE, the remaining part of that particle continues from the opposite side. For each mesh element, the properties were averaged according to the imported density map, and the effective properties of the entire macrostructure were calculated based on the nanoscale model. Properties obtained from the nanoscale-averaged results were then assigned to the grid representing graphene in the overall nanocomposite RVE model using the properties listed in Table 5.

From molecular dynamics simulations, the averaged values for the thickness of the nano-reinforcement used in the FEA model are shown in Table 6. Equilibrium thickness (t), radius, Young’s modulus (E), and the in-plane tensile rigidity (Y) were obtained using the Irving–Kirkwood averaging calculations of displacement properties.

The predictions of representative macroscopic properties of graphene epoxy nanocomposites considered in this work were the final step of the multiscale modeling strategy. FEA calculations were performed to analyze the overall nanocomposite properties. The density profiles obtained from nanoscale simulations were mapped to a fixed cubic grid in a macroscale model. The resulting effective properties were estimated using an energy minimization method. Figure 9a shows a 3D cubic RVE filled with graphene nano-reinforcement with an aspect ratio of 100. Figure 9b shows RVE with more flexible meshing, and each disc was partitioned into four symmetric parts.

## 5. Results

The multiscale modeling results are discussed starting with the functionalization of graphene using OH and COOH functional groups of 3%, 6%, and 9.8% respectively. The functionalized graphene with improved properties was determined by the valence of the oxygen atoms attached to the functionalized graphene sheet. Subsequently, a functionalized graphene sheet with an epoxy matrix was developed to obtain the interphase properties using the MD modeling technique. The different weight fractions of graphene sheets were attached to the epoxy matrix, and the best weight fraction was obtained by checking the cross-linking between the graphene sheet and the epoxy matrix. The modeled graphene epoxy nanocomposites were then tested to obtain the interphase properties by applying a displacement (normal and shear direction) to the graphene sheet. As the displacement was applied, the traction forces were extracted to characterize the interphase properties of the developed nanocomposites. The MD-modeled nanocomposite properties were transferred to the macroscale model by coupling the two scales. The coupled models were then analyzed to obtain the elastic properties of the graphene epoxy nanocomposite. The results were then validated using the available literature.

### 5.1. Graphene Functionalization

**Figure 10:**
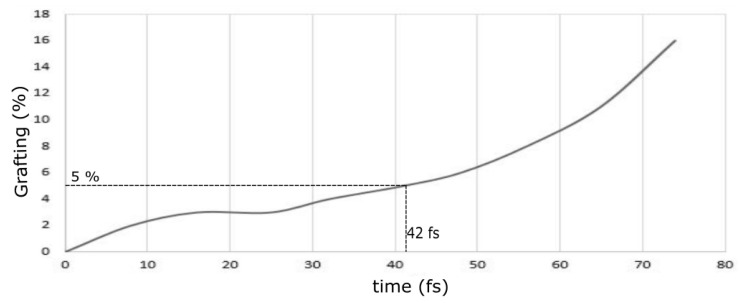
Graphene functionalization grafting percentage with time.

Figure 10 shows the increasing functional group grafting with increasing time. As the time increased, bonds were formulated between the graphene sheet and the functional groups, with oxygen and hydrogen atoms creating covalent bonds with carbon atoms up to 73 fs. As the functionalization process began, there was a gradual increase in grafting percentage up to 42 fs. This is because, after 5% grafting, the graphene sheet showed signs of saturation in functionalities owing to the number of valence oxygen atoms attached during functionalization.

The Young’s modulus and strength of the functionalized graphene sheets were calculated using the minimization commands by varying the LJ parameters. The results are given in Table 7 along with the comparative experimental results [33,34]. The Young’s modulus and tensile strength of the 3% grafted graphene sheet were very low compared with the 6 and 9.8% grafted graphene. However, the 6% grafted graphene sheets showed mechanical properties (E = 0.89 TPa and strength = 121 GPa) that align with the experimental data of E = 1.00 ± 0.1 TPa and strength = 130 ± 10 GPa and thus were selected to improve the epoxy properties within the nanocomposite.

### 5.2. Crosslinking of Functionalized Graphene and Epoxy

**Figure 11:**
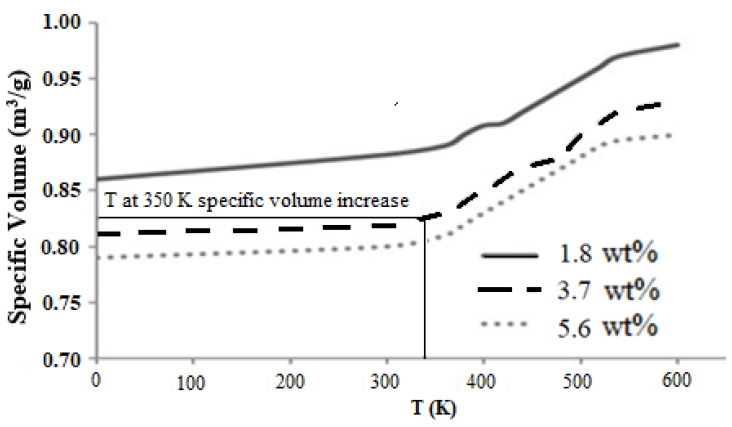
Specific volume vs. temperature for epoxy nanocomposite.

The functionalized graphene sheet was then cross-linked with an epoxy matrix to analyze the interfacial properties. First, the graphene epoxy unit cell was equilibrated at room temperature of 350 K to reach a balanced state, as the density of the unit cell fluctuates in a small range around the target temperature. The equilibration was carried out by controlling the NVT parameters, which provided the temperature versus specific volume curves. These curves define the cross-linking behavior of epoxy graphene nanocomposite and are shown in Figure 11. The cross-linking started with one graphene sheet (1.8 wt.%), and the specific volume of the unit cell was constant at low temperatures, as there was no change in thermal properties (expansion). At 350 K (the transition temperature), the specific volume started to increase rapidly owing to expansion of the unit cell as the temperature increased further. The specific volume at this transition temperature for one graphene sheet was 0.79 cm^3^/g, showing incomplete cross-linking within the unit cell. As the nanocomposite approached higher cross-linking temperatures, the specific volume increase was more gradual and the unit cell united atoms packed closely to each other.

**Figure 12:**
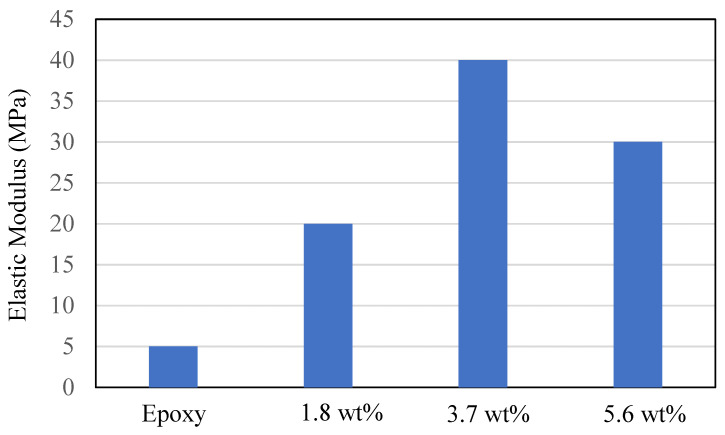
Elastic Modulus with the addition of graphene sheets.

Further addition of two graphene sheets (3.7 wt.%) showed perfect bonds, which were shown by the specific volume at a transition temperature of 0.82 cm^3^/g. The cross-linking of two graphene sheets with the epoxy matrix showed improvement in the formation of covalent bonds between the united atoms, thereby demonstrating strong cross-linking. The addition of the third graphene sheet (5.6 wt.%) showed saturation in cross-linking of the united cell atoms at a high specific volume of 0.89 cm^3^/g. The reason behind this behavior is that at this point, the number of valence united atoms increased, which means that the van der Waal forces were higher between the united atoms due to the unbonded atoms within the unit cell. The transition temperature (at 350 K) was the corresponding temperature at the slight inflection point of the slopes of specific volume versus temperature linear curves. Therefore, a perfect weight fraction was determined for the graphene epoxy nanocomposite, which proved to be two graphene sheets (3.7 wt.%). Each layer of graphene sheet added to the epoxy matrix increased the mechanical properties of the nanocomposite. The elastic modulus increased with the addition of each graphene sheet, and two graphene sheets (3.7 wt.%) achieved the highest modulus. Adding a third graphene sheet resulted in saturation in mechanical properties, as shown in Figure 12.

### 5.3. Interphase Region Properties

**Figure 13:**
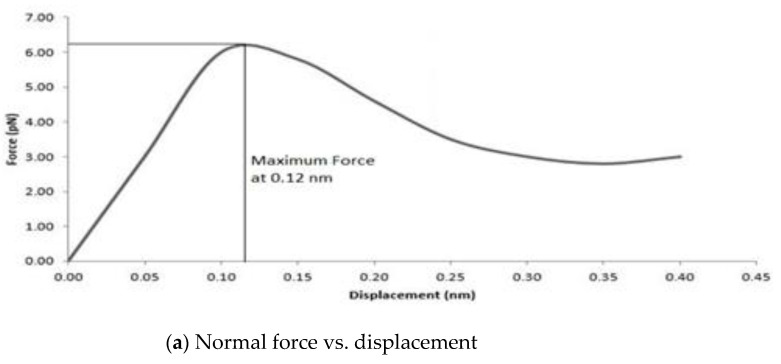

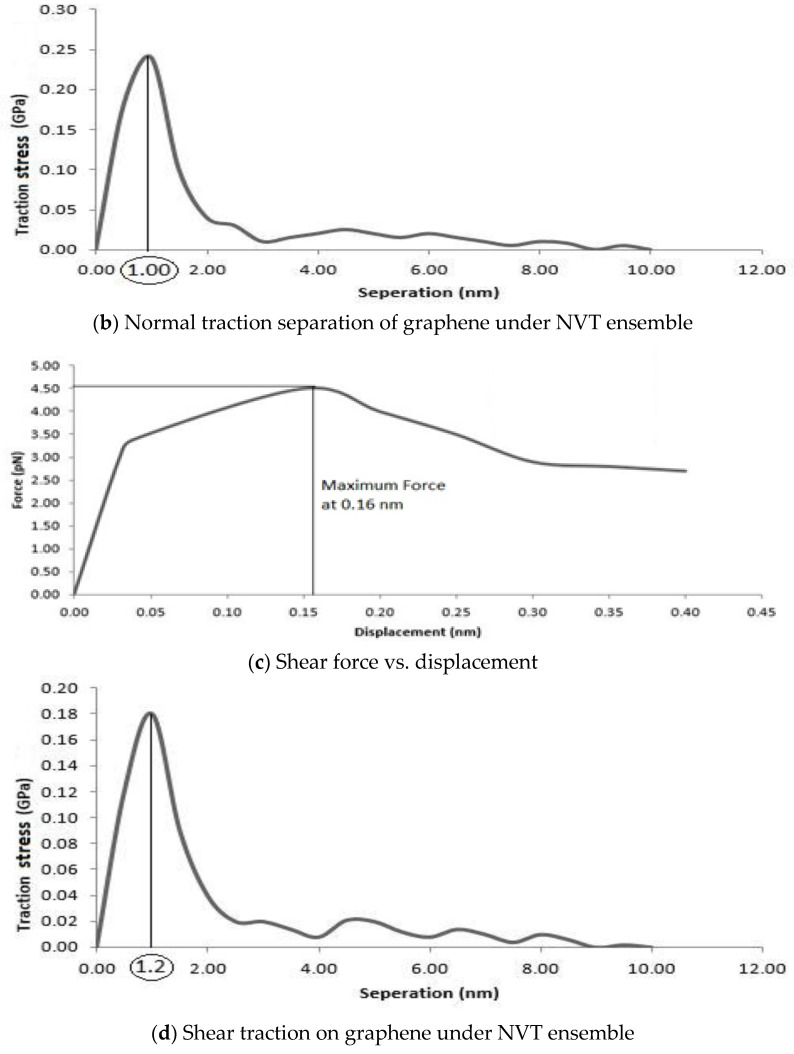
Interphase region characterization.

After the unit cell was equilibrated, the graphene sheet was then displaced to obtain the interphase region properties along the normal and shear directions. The displacement applied along the normal direction of graphene helps to explain the pullout mechanism between graphene and epoxy. As the graphene sheet was pulled under the displacement control, a reaction force was developed at the interface between the graphene sheet and the matrix. Figure 13a shows the force-displacement curve, which is linear at the initial stage and reaches a maximum force of 6.2 pN at a displacement of 0.12 nm. The smaller magnitude of the normal force is due to the van der Waals force interaction between the graphene and epoxy. Such normal force may be increased by minimizing the gap between the graphene and epoxy or introducing more covalent bonds between the graphene and epoxy.

From the pullout test, the traction-separation relationships for graphene and epoxy were obtained and plotted as shown in Figure 13b. This was achieved by measuring atom-by-atom separation as a function of traction. The traction stresses were calculated by dividing the traction force by the initial section area of the composite (equal to an effective graphene area). The traction strengths increased up to 1 nm displacement and then started to decrease. The values were approximately constant from 2 nm to 10 nm. From 2.5 nm, the traction curves show significant fluctuations, and this indicates that 2.5 nm is the point where the number of interfacial bonds results in a converged response for the separation of the graphene sheet from the epoxy matrix.

Figure 13c shows the shear force versus displacement curve at the interfacial region for the graphene epoxy nanocomposite. The maximum force and displacement for shear force are 4.5 pN and 0.16 nm, respectively. This demonstrates a nonlinear behavior before reaching a peak point. The shear force then gradually drops with increased displacement. The shear traction separation is shown in Figure 13d, with the peak value at 1.2 nm. The peak value of traction was the critical fracture stress at which crack initiation occurred, and it directly corresponded to the highest traction stress. Further increase in separation led to a decrease in traction, eventually reaching a minimum at 8.3 nm. The area under the curve is equal to the energy needed for separation.

### 5.4. Characteristics of the MD Models

To understand the characteristics of the simulated molecular models, the radial distribution function (g(r)) and molecular energy were analyzed.

#### 5.4.1. Radial Distribution Function (RDF)

**Figure 14:**
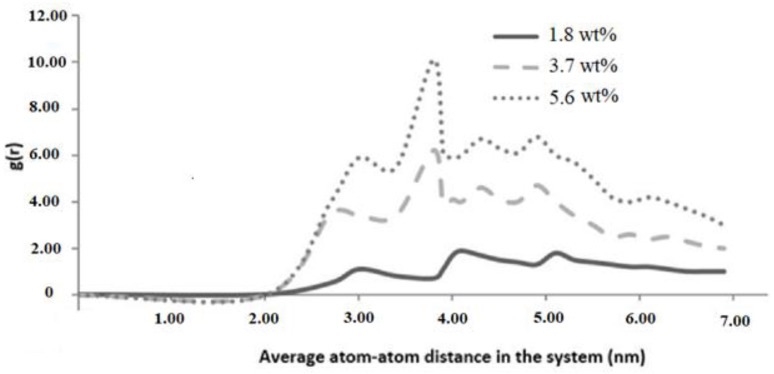
RDF pattern for graphene and epoxy particles in epoxy nanocomposite.

The RDF was obtained directly from the MD simulations and provided a distribution pattern of graphene atoms and epoxy molecules within the epoxy nanocomposite with the addition of 1.8, 3.7, and 5.6 wt.% graphene. Figure 14 shows the RDF for the graphene epoxy nanocomposite systems. The absence of sharp peaks in the RDF ensured the amorphous nature of the graphene–epoxy system. The highest peak for 3.7 and 5.6 wt.% graphene reinforcements was observed at 3.9 A°, which indicated the maximum concentration of atoms in the entire system at this pairwise distance. The influence of graphene concentration was seen to be insignificant on RDF for 1.8 wt.% graphene epoxy nanocomposite. However, the highest and lowest values of RDF were seen in epoxy nanocomposite with 3.7 and 5.4 wt.% of graphene concentrations with very similar phenomena. Such observations are possibly due to insignificant variations in densities. These peaks correspond to carbon atoms that are connected by one or two bonds within the system. The contribution of carbon–carbon bonds from both graphene structures is also attributed to this peak.

#### 5.4.2. Bond Stretching and Angle Bending during Deformation

**Figure 15:**
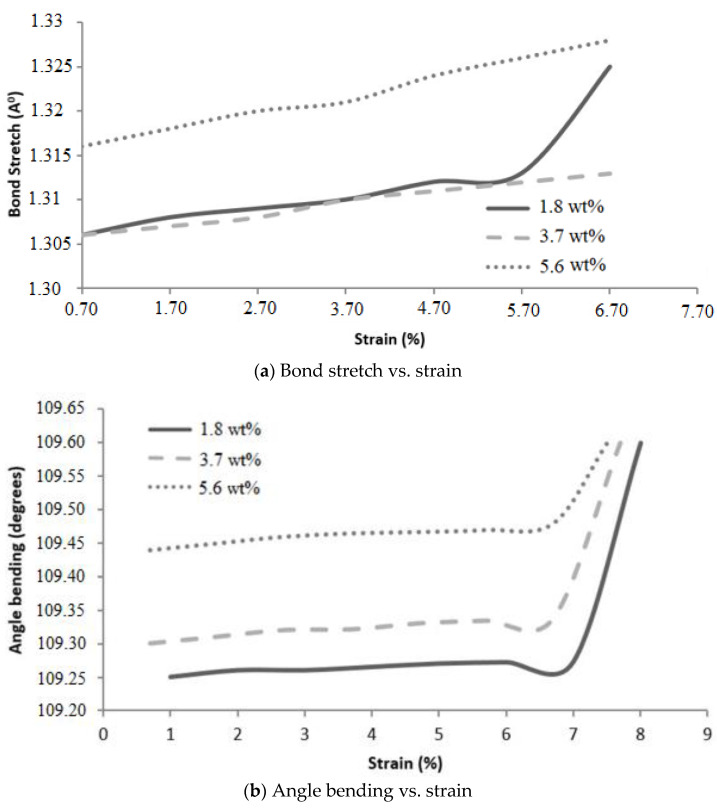
Molecular bond behavior during deformation.

The bond stretch and bond angle play important roles in predicting the stiffness of the nanocomposite; therefore, the pairwise bond stretch and angle bend due to applied strain were determined, as shown in Figure 15. As the atoms undergo local relaxation between 0% strain and the first deformation, the data up to the first deformation were ignored. As shown in Figure 15a, the epoxy nanocomposites with 1.8 wt.% and 3.7 wt.% graphene have very similar slopes, although the average pairwise bond deformed comparatively faster with 3.4 w.t.% graphene. The average bond stiffness for 5.6 wt.% graphene epoxy nanocomposite is slightly higher than the other two nanocomposite configurations, and the stiffer pairwise bonds in 5.6 wt.% resulted in less stretch in the bond length. Similar behavior was observed for the angle bending with increasing strain (Figure 15b). For the epoxy nanocomposite with 3.7 wt.% graphene, the average pairwise bond angle rates to applied deformation were almost similar to the 1.8 wt.% graphene epoxy nanocomposite. These curves are almost parallel with different initial bending angles, which is consistent with the stress–strain response and potential energy evolution for these unit cells.

#### 5.4.3. Molecular Energy

**Figure 16:**
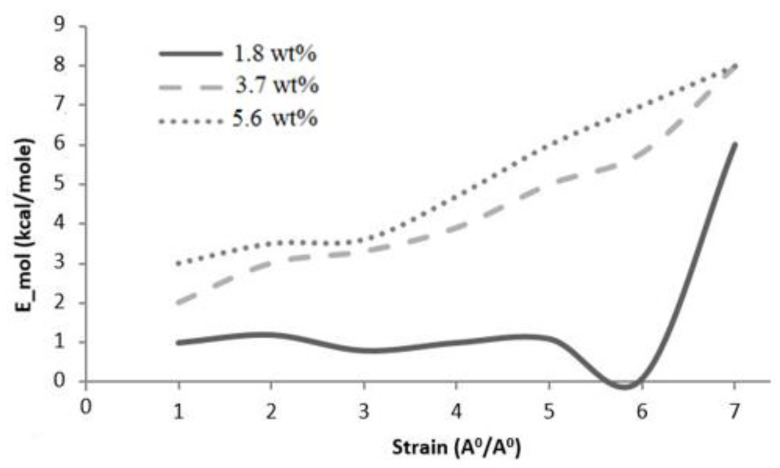
Molecular energy variation with strain for graphene epoxy nanocomposites.

The displacement of the graphene sheet in the MD simulations caused a change in atom positions, velocities, and overall molecular structure, thereby increasing overall potential energy. The potential energy made a comparatively larger contribution to total molecular energy than van der Waals energy. The change in molecular energy followed by applied deformation indicated the sensitivity of the molecules to applied strain. Molecular energy versus strain plots for graphene epoxy nanocomposites with 1.8, 3.7, and 5.6 wt.% graphene are shown in Figure 16. The molecular energy for a unit cell with 1.8 wt.% graphene showed gradual fluctuations in molecular energy, possibly owing to the lower elastic modulus provided by 1.8 wt.% graphene. The increase in the slope of molecular energy then clearly explained the deformation in the molecular topology with applied displacement. The molecular energy curve of 3.7 wt.% graphene is comparatively steeper and higher, indicating a higher modulus than 1.8 wt.% of graphene. This increase in slope implies a higher modulus provided by the increase in the number of graphene sheets. The change in molecular energy is observed to be very small for 5.6 wt.% graphene compared with 3.7 wt.% graphene. 

### 5.5. Macroscale Properties of the Graphene Epoxy Nanocomposites

**Figure 17:**
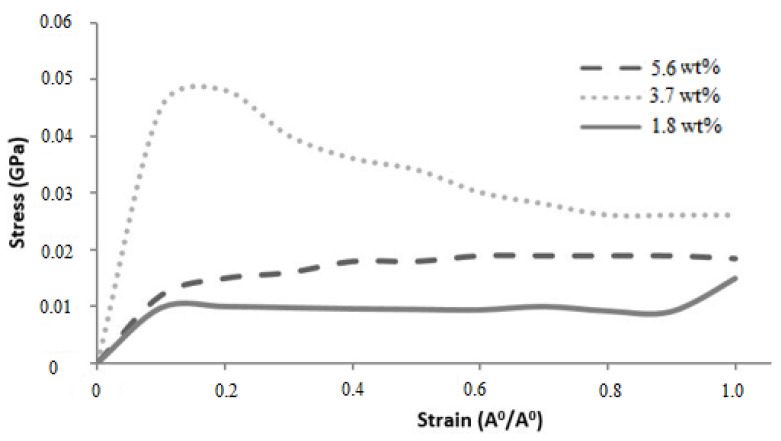
Stress–strain responses of graphene sheet nanocomposites.

The simulated stress–strain responses of epoxy nanocomposites with graphene concentrations of 1.8, 3.7, and 5.6 wt% are shown in Figure 17. The Young’s modulus (E) values were determined from the slopes of individual curves and are given in Table 8. Fluctuations in the stress–strain responses were minimized by applying the moving average technique. The elastic moduli of the nanocomposites with 1.8 and 3.7 wt.% graphene were comparatively higher than the 5.6 wt.% graphene reinforcement. The reduction in elastic modulus for the increase in graphene reinforcement is due to the distribution of atoms, angle stretch, angle bend, and change in molecular energy in the atomistic model unit cell, which plays an important role in defining the properties. Hence, increasing Young’s modulus with respect to the weight fraction of graphene sheets may not be realistic in the actual scenario. It is noted that the experimentally calculated Young’s modulus [35] shows higher values with increased graphene concentrations, but in numerical simulations, increasing the graphene sheet to 3.7 wt.% increased the nanocomposite properties, and further increased graphene weight fractions reduced the properties of the nanocomposite system.

**Figure 18:**
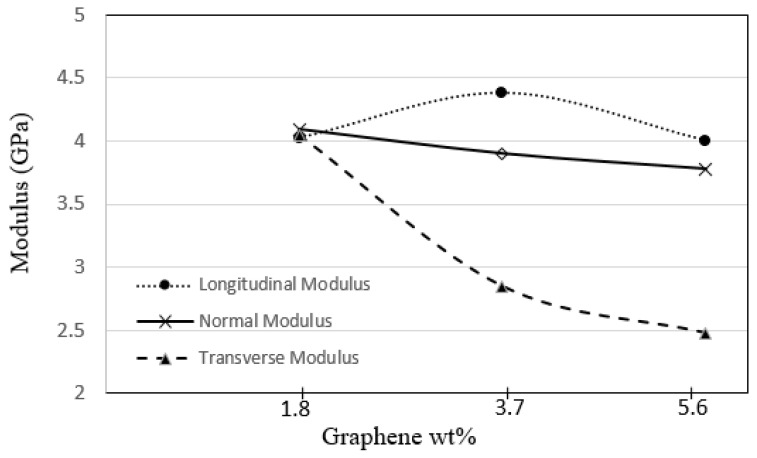
RVEs modulus for the increasing graphene weight fractions.

The variation in macroscale properties of the graphene epoxy nanocomposites with the addition of different weight fractions of numbers of graphene sheets is shown in Figure 18. The longitudinal modulus increased constantly with an increase in the number of graphene sheets. For the 1.8 wt.% graphene, the peak value of the longitudinal modulus is at 4.02 GPa, and with the further addition of 3.7 wt.% graphene, the longitudinal modulus (E_x_) increased to 4.38 GPa. This shows that the addition of the graphene nano-reinforcement increased the mechanical properties of the epoxy nanocomposites. Similar behavior was observed for the normal modulus (E_y_), which increased with the addition of 1.8 wt.% graphene and then plateaued for further increases in graphene reinforcements. However, the transverse modulus (E_z_) increased to 4.05 GPa with the addition of 1.8 wt.% graphene, and the properties drastically reduced with further addition of graphene sheets. This shows that the transverse direction was not affected by increasing the graphene sheets. Therefore, 3.7 wt.% of graphene results in improved mechanical properties for the graphene epoxy nanocomposite.

### 5.6. Validation of Numerical Results Using Available Literature Data

The mean value of the stiffness of numerically tested samples of 1.8, 3.7, and 5.6 wt.% graphene and the stiffness obtained from experimental results from the literature [36,37,38] were compared (refer Table 9). The logarithmically extrapolated experimental results are lower than the multiscale model results because the experiments cannot replicate the molecular scale behavior. However, the MD results align with the experimental results of Wan et al. [36,37]. The reason behind the close match might be the attachment of the functional group to the graphene via silane functionalization. As per the cited paper, the silane functionalization has an attached OH and COOH functional group, which is similar to the attached functional group modeled in MD analysis. This validates the results obtained from the multiscale modeling of graphene epoxy nanocomposites.

## 6. Conclusions

Graphene epoxy nanocomposite was characterized using a multiscale modeling technique. The MD modeling was used to analyze the effect of graphene on polymer composite interphase properties. Molecular dynamics was coupled with finite element analysis using the Irving–Kirkwood method. The extracted properties (boundary conditions, initial conditions, and mechanical properties) were used to model the finite element model and analyze the macroscale properties. The multiscale modeling started with the functionalization of graphene using 3%, 6%, and 9.8% OH and COOH functional groups, respectively. The Young’s modulus and tensile strength of the 6% grafted graphene sheet were most closely comparable to the experimental results and were thus selected for improving the epoxy properties within the nanocomposites. The functionalized graphene was then cross-linked with an epoxy matrix to determine the interphase properties. Three weight fractions of graphene sheets (1.8, 3.7, and 5.6 wt.%) were attached to the epoxy matrix, and the best weight fraction was obtained by checking the cross-linking between the graphene sheet and the epoxy matrix. Using the transition temperature of 350 K, the slopes of the specific volume versus temperature were used to determine the perfect weight fractions for the graphene epoxy nanocomposites. The results showed that the graphene with 3.7 wt.% achieved the highest modulus. Subsequently, a functionalized graphene sheet with an epoxy matrix was developed to obtain the interphase properties using the MD modeling technique. The modeled graphene epoxy nanocomposites were then tested to obtain the interphase properties by applying a displacement (normal and shear direction) to the graphene sheet. As the displacement was applied, the traction forces were extracted to characterize the interphase properties of the developed nanocomposites. The MD analysis results were analyzed using RDF, bond stretching, angle bend, and molecular energy. The radial distribution function maximum concentration was observed at an approximate pairwise separation distance of 3.9 Å and was only slightly distinguishable for the smaller 1.8 wt.% of graphene. The pairwise bond lengths of the 3.7% and 5.6 wt.% graphene-based systems were slightly higher than the 1.8 wt.% graphene system. Further, the slope of the molecular energy versus strain plots showed progressive deformation in the graphene epoxy nanocomposite system and resulted in increased elastic moduli of the graphene epoxy nanocomposite. For the graphene epoxy nanocomposite model, the solution was obtained by minimizing the energy function. The simulated stress pairwise bond lengths strain properties from the heterogeneous MD coupled FEA mode showed that the elastic moduli of the nanocomposites with 1.8 and 3.7 wt.% graphene were comparatively higher than the 5.6 wt.% graphene reinforcement. The reduction in elastic modulus with the increase in graphene reinforcement is due to the distribution of atoms, angle stretch, angle bend, and change in molecular energy in the atomistic model unit cell, which plays an important role in defining the properties. Hence, increasing Young’s modulus with respect to the weight fraction of graphene sheets may not be realistic in the actual scenario. Further analyzing the three different moduli, the longitudinal and normal moduli increased with the addition of 1.8 wt.% graphene and then plateaued with further addition. However, the transverse modulus increased for the 1.8 wt.% graphene and then reduced drastically with further increases in graphene reinforcement. The macroscale mechanical properties of the graphene epoxy nanocomposite closely align with the literature data.

## Figures and Tables

**Table 1:** MD initial setup for epoxy matrix modelling.

MD Model Setup	DGEBA and DETDA Hardener (Epoxy)
Stoichiometric ratio	2:1
Unit cell size	10 × 10 × 10
Boundary conditions	Periodic
Force Field	OPLS (Optimized Potentials for Liquid Simulations)
Cut-off radius	10 Å
Total number of atoms	EPON 862 = 31 DETA = 21

**Table 2:** MD initial setup for graphene modeling.

MD Model Setup	Graphene Sheet
Units	Metal (atomic style)
Unit cell size	4 × 4
Boundary conditions	Periodic
Interatomic Potentials	Tersoff
Ensemble	NVT (T = 300 K)
Total number of atoms	32 Carbon atoms

**Table 3:** Change in number of atoms during functionalization.

	Functional Groups Percentage (%)	Number of Atoms Added by Functional Groups	The Total Number of Atoms in the Graphene Sheet
Graphene sheet (32 atoms)	3.0	7	39
	6.0	14	46
	9.8	23	55

**Table 4:** Graphene weight fraction and material configuration of the nanocomposites.

Model	Epoxy United Atoms	Graphene United Atoms	Weight Fraction (wt%)	Unit Cell Dimension (Å)
1 graphene nanocomposite	52	1	1.8	a = 9.84, b = 19.02, c = 1056.42
2 graphenes nanocomposite	52	2	3.7	a = 9.84, b = 19.02, c = 2116.24
3 graphenes nanocomposite	52	3	5.6	a = 9.84, b = 19.02, c = 3104.21

**Table 5:** The nanocomposite properties extracted from the nanoscale model.

Properties	Graphene Epoxy Nanocomposite
Young’s modulus(E)	4.56
Weight fraction (%)	3.77
Poisson’s ratio (v)	0.36

**Table 6:** Averaged properties for FEA modeling.

Radius (cm)	E (TPa)	t (cm)	Y (TPa)
2.5	3.74	0.142	0.329
5.0	3.80	0.141	0.330
9.5	3.76	0.141	0.327

**Table 7:** Validation of MD results using experimental data.

Graphene Sheet Properties	Young’s Modulus E (TPa)	Strength (GPa)
3% grafted sheet	0.64	98.3
6% grafted sheet	0.89	121
9.8% grafted sheet	1.53	127
Experimental results [33,34]	1.00 ± 0.1	130 ± 10

**Table 8:** Young’s modulus of graphene epoxy nanocomposites.

Material Configuration	Weight Fraction (%)	Young’s Modulus E (GPa)
One Graphene Sheet	1.8	4.02
Two Graphene Sheets	3.7	4.38
Three Graphene Sheets	5.6	4.01

**Table 9:** Comparative analysis of the MSM with logarithmically extrapolated experimental results for epoxy reinforced with functionalized graphene.

Graphene wt.%	MSM Model: Longitudinal Modulus (GPa)	Elastic Modulus (GPa)			
		[35]	[36]	[37]	[38]
1.8	4.02	2.91	3.88	3.94	2.90
3.7	4.38	2.96	4.04	4.08	2.98
5.6	4.01	3.09	4.13	4.16	3.02

## Data Availability

Data are contained within the article.
